# Physician agency in China: evidence from physicians’ responses to financial pressure during the COVID-19 pandemic

**DOI:** 10.1186/s13561-025-00692-x

**Published:** 2025-11-27

**Authors:** Qi Zhang

**Affiliations:** 1 Cumming School of Medicine, University of Calgary, Calgary, Canada; 2 Canadian Centre for Health Economics, Toronto, Canada

**Keywords:** Physician agency, Financial incentives, Healthcare utilization, China, Rural health

## Abstract

This paper examines how rural primary care physicians in China adjusted their practice patterns to pandemic-related financial pressures under a capitated global-budget model. Using township-hospital data, we find increased prescribing of Traditional Chinese Medicine (TCM) decoction pieces, with effects concentrated among habitual prescribers rather than converting occasional users into regular prescribers. Physicians also reduced both the number of drugs prescribed and the volume of services provided to cost-sharing outpatients, producing a 5% decline in average insurance payments per outpatient visit and potentially generating a greater surplus within the global-budget pool. By contrast, we observe no significant changes for self-paying outpatients, suggesting limited scope for physician-induced demand. These results underscore the role of physician agency in healthcare provision and highlight the importance of aligning financial incentives with policy goals. While drug reforms and managed-care models have contained expenditures, challenges remain in achieving adequate coverage for rural residents.

## Introduction

The rapid growth of total health expenditure has become a common concern for most countries [1, 2]. In China, total health expenditure has grown considerably since the start of economic reforms in 1978, consistently outpacing GDP growth [3]. The problem of rising health care expenditure is partially attributed to physicians, who play a central role in delivering health care [4]. Many resources in the health care system are directed by physicians that in turn determine costs and health outcomes for patients.

Since a seminal contribution by Arrow [5], the role of physicians’ motives and their influence on the care patients receive has been recognized as a central issue in health economics, commonly described as “physician agency” [6]. In this framework, “agency” denotes the physician’s capacity to act on the patient’s behalf [7]. However, physicians may also leverage this relationship to steer patient preferences in ways that advance their own interests—physician-induced demand (PID) [6].

Understanding physician agency across diverse settings is essential for designing policies to contain health-care spending. Although there is a substantial theoretical and empirical literature on the topic (for reviews, see [6, 8]), evidence from low- and middle-income countries (LMICs) remains limited.

Physicians working in rural settings of LMICs often face different incentives compared to their counterparts in developed economies. In rural China, public township hospitals serve as the main provider of primary health care [9]. Physicians in these hospitals are salaried employees, but a significant portion of their income comes from bonuses tied to the hospital’s net revenue [10].

Despite the lifting of COVID-19 lockdown restrictions in most provinces beginning in March 2020, outpatient and inpatient volumes at township hospitals had not returned to pre-pandemic levels by early 2021, largely because patients remained concerned about contracting the virus when seeking care [11]. The sharp decline in service volumes reduced hospital revenue [12], with likely negative effects on physicians’ income. The drop in inpatient volume was larger than the decline in outpatient visits in township hospitals [11]. Because township hospitals primarily provide outpatient care and offer only a limited range of inpatient services, physicians’ behavioral responses were more likely to appear in outpatient settings; unlike in tertiary hospitals, they had limited scope to induce demand among hospitalized patients [13].

To provide new empirical evidence on physician agency, this paper examines how physicians respond to the financial pressures of reduced hospital revenue during the COVID-19 pandemic. Specifically, we compare physicians’ practice patterns in outpatient care before and after the COVID-19 outbreak to understand how they react to incentives within the current policy context in rural China. Prices of drugs and medical services in township hospitals are regulated by the government. Since 2009, the Chinese government has mandated zero markups on drugs sold in public hospitals, with the exception of Traditional Chinese Medicine (TCM) decoction pieces—raw or processed herbs, such as roots, leaves, or minerals—that are cut and prepared for making herbal teas or decoctions used in TCM treatments [14].

Additionally, there are two types of outpatient visits based on the payment method: (1) cost-sharing visits, where patients pay a co-insurance fee, with the remaining costs covered by the public insurance program through insurance payments; and (2) self-paying visits, where patients pay the full price out-of-pocket. Both types of visits are billed under the same government-regulated fee schedule on a fee-for-service basis. Notably, township hospitals operate under a capitated global budget (CGB) system for outpatient care, which covers the insurance payments for all cost-sharing visits during the contract year. At the end of the year, public insurance authorities compare the total incurred insurance payments against the allocated global budget. Any surplus in the budget is distributed to the hospital as a bonus, a significant portion of which is paid to physicians as part of their income.

Although both self-paying and cost-sharing visits are subject to the same fee schedule and are billed on a fee-for-service (FFS) basis, physicians’ incentives differ between the two. While self-paying visits are treated as traditional FFS encounters, cost-sharing visits are more likely to align with the incentives embedded in the CGB scheme.

To study physician agency under the incentive schemes imposed by the current policy context, it is important to distinguish between the payment methods of outpatient visits and analyze them as two distinct samples. To examine how physicians responded to the financial pressures caused by the COVID-19 pandemic, we acquired a micro-level dataset of outpatient visits from township hospitals in two rural counties in a southern province of China. This dataset includes exhaustive records from a 12-month period before the COVID-19 outbreak (January 2019 to December 2019) and a 12-month period after (March 2020 to February 2021). Each outpatient visit record contains information such as the visit date, attending physician, diagnosis, drugs dispensed, total expenditures, drug and non-drug service expenditures, insurance payments, and out-of-pocket expenses. It also includes patient characteristics like age, sex, and payment method (cost-sharing or self-paying).

In response to reduced hospital revenue, as noted earlier, physicians may have tried to maintain their incomes by inducing patient demand for Traditional Chinese Medicine (TCM) decoction pieces, for which up to a 25% markup was allowed (unlike Western medicines and TCM patent medicines, for which no markup was permitted). The first research question investigates to what extent physicians responded by increasing prescriptions of TCM decoction pieces for cost-sharing and self-paying visits during the COVID-19 pandemic, compared to before the pandemic.

The second research question explores the heterogeneity of physician agency based on the payment method of the outpatient visits. During the pandemic, the surplus from the capitated global budget (CGB) fund, which is distributed as bonuses, became increasingly important for physician income. Consequently, for cost-sharing visits during the pandemic, it is hypothesized that physicians would reduce insurance payments per visit. Since insurance payments are positively linked to total expenditures, this would likely result in lower health expenditures (both for drugs and non-drug services) compared to similar visits before the pandemic.

In contrast, for self-paying visits, which follow a pure fee-for-service (FFS) model, increasing non-drug services is the only way to boost hospital net revenue and, ultimately, physician income, under the zero-markup policy on drugs. However, physicians may not be able to induce more non-drug services for self-paying visits if the demand for treatment is primarily driven by patient preferences [15, 16]. On the one hand, township hospitals offer only general consultations and basic diagnostic tests, limiting the range of services available for demand inducement. On the other hand, rural self-paying patients are often financially constrained and may not be able to afford additional services. For these reasons, the decline in self-paying visits during the pandemic may not lead to an increase in non-drug services per visit in rural settings.

Using a before-and-after empirical strategy that controls for patient characteristics and physician fixed effects, we find that physicians were significantly more likely to prescribe TCM decoction pieces during the pandemic for both self-pay and cost-sharing visits, relative to comparable pre-pandemic visits. The increase was concentrated among habitual prescribers, with only modest changes among occasional prescribers. Additionally, our analysis reveals that insurance payments, as well as drug and non-drug service expenditures, decreased significantly for cost-sharing visits after the COVID-19 outbreak compared to the pre-pandemic period. In contrast, we did not observe a significant change in non-drug service expenditures for self-paying visits, suggesting that demand for these services is largely driven by patient preferences and/or that physicians have limited opportunities to induce additional demand. These findings are broadly consistent with the predictions of agency theory.

The remainder of the paper is organized as follows. The Related literature section highlights the contributions of this study in relation to other studies in the literature. The Background section provides an overview of the institutional setting. The Conceptual framework section outlines the theoretical foundations and formulates the testable hypotheses. The Data section describes the data sources and key variables, while the Empirical approach section details the estimation methods. The Results section presents the main findings, followed by the Discussion and Conclusions section, which summarizes the implications and concludes the paper.

### Related literature

This paper contributes to the empirical literature on physician-induced demand (PID)—the idea that physicians may provide services with low marginal benefit to increase income [17]. Early studies examined the relationship between physician density and service volume [4, 18, 19]. For example, a 10% increase in the surgeon-to-population ratio was associated with a 3% increase in surgeries per capita, suggesting that physicians offset declining patient volumes with more intensive treatment [4]. Similarly, subsequent work reported that higher physician density increased service provision and overall costs [18, 19].

Using fertility-rate declines as an instrument for physician supply, researchers showed that physicians substituted less profitable vaginal deliveries with higher-paying Cesarean sections in response to income shocks [20]. In France, as the physician-to-population ratio rose, physicians provided more services per consultation [21]. However, Norwegian studies found no evidence of PID, despite similar regulations and fee structures [22–24].

Our study adds to this literature by leveraging patient-level data from rural China, rather than relying on geographic or physician-level aggregates. We exploit exogenous variation in patient volume due to the COVID-19 pandemic to assess changes in physician behavior.

This paper also contributes to research on physician agency in China. In a field experiment, physicians prescribed more expensive medications to insured patients [25]. Informing physicians about external prescription filling reduced antibiotic overuse [26].

Prior to the national zero-markup drug policy, a drug-expenditure cap in tertiary hospitals led physicians to substitute from drugs to services—especially for insured patients—in order to maintain revenue [16]. During the staggered rollout of the zero-markup policy, difference-in-differences analyses using inpatient records from township and county hospitals showed that drug expenditures declined while non-drug services increased, largely offsetting the policy’s financial effects [27].

We extend this literature by examining physician agency in the outpatient setting under a capitated global-budget regime after full implementation of the zero-markup policy. Township hospitals are key providers of outpatient care in China, yet the financial incentives they face differ markedly from those in urban tertiary hospitals and have previously received limited study. Moreover, public insurance for rural residents is less generous than employer-based coverage for urban workers, which may shape physician agency—including induced demand—in ways that differ from urban settings.

Finally, we complement studies on health service utilization during COVID-19 in China [11, 12, 28–30], which document widespread reductions in care. We focus on how primary care physicians in rural areas responded to these utilization shocks, considering financial incentives under current policy frameworks.

## Background

### Regional COVID-19 conditions

From March 2020 to February 2021, no COVID-19 cases were reported in the study region.^1^ However, patients with symptoms like fever were restricted from seeking care at township hospitals and redirected to designated tertiary hospitals. Many patients also delayed or avoided in-person care during this period [31].

### Township hospitals in the region

Township hospitals are government-owned, non-profit facilities providing primary and outpatient care. In 2019, the province had 1,180 township hospitals with an average of 61,757 outpatient visits. However, in the study counties, outpatient volume was lower, averaging about 15,000 visits per hospital. During the pandemic year, outpatient visits dropped by 18.1%, mirroring the provincial decline [32]. These hospitals lacked a unified telemedicine system, and declining patient volume reduced revenue, creating financial pressure [33].

### The 2009 drug reform

To contain rising drug costs, China introduced a 2009 reform consisting of the National Essential Medicines List (NEML), a zero markup policy (ZMP), and centralized procurement. The 2018 NEML includes Western medicine, TCM patent drugs, and TCM decoction pieces [27, 34]. The ZMP eliminated markups on most drugs except TCM decoction pieces. Drug prices are set through provincial bidding, standardizing costs across hospitals. These policies have been in effect nationwide since 2017.

### The public insurance program in rural China

The “Urban and Rural Resident Basic Medical Insurance” (URRBMI) covers rural and unemployed urban residents. It is voluntary and funded by fixed annual premiums, which account for approximately 30% of the total cost, with the government subsidizing the remaining 70%. Since 2015, over 95% of the rural population has been covered [35]. URRBMI is managed at the prefecture level,^2^ and enrollees must select a contracted township hospital annually. Outpatient coverage is available only at or via referrals from the contracted hospital, with coinsurance rates of 30–40% and no deductible up to a capped limit.

### The incentive scheme for health providers

In self-paying visits, hospitals receive payments directly from patients. Under URRBMI, outpatient services are covered by a capitated global budget based on the number of contracted enrollees. Hospitals receive 80% of the annual budget in monthly installments, with the remaining 20% withheld until year-end reconciliation. If actual surplus is within 20% of the annual budget, the surplus is paid to the hospital as bonuses. If the surplus exceeds 20%, the excess is forfeited as a penalty. If spending exceeds the budget, the hospital bears the financial loss. Physicians are salaried employees of hospitals [36]. Their compensation comprises a base salary and benefits, plus bonuses tied to hospital net revenue, which often represent a substantial share of total income [10, 37, 38]. While base pay is government-regulated, bonus schemes are set by individual hospitals and typically account for 30% to 40% of physician income, though the share can be higher or lower depending on hospital operating results [39].

## Conceptual framework

This section outlines the conceptual framework for analyzing physicians’ behavior in care provision in response to financial implications during COVID-19. The reduction in patient volume imposed financial pressure on hospitals, which in turn affected physicians’ income. To mitigate this negative influence, physicians may have adjusted their practice patterns for outpatient visits during the pandemic. Specifically, physicians could increase the net revenue of the hospital they work for by providing more care with a positive mark up for self-paying visits and/or decrease the care provided for cost-sharing visits to create a surplus in the capitated global budget fund.

After the fully implementation of zero-markup policy, drugs sold at township hospitals had to be provided with no markup, except for TCM decoction pieces, which could have up to a 25% markup. Given the fee-for-service nature of self-paying visits, an increased prescription of TCM decoction pieces for these visits would have been more advantageous to physicians. For cost-sharing visits, physicians could also increase prescription of decoction pieces following the COVID-19 outbreak. Since decoction pieces are relatively niche, increased prescribing was unlikely to result in significantly higher average drug expenditure for cost-sharing visits. The unique characteristics of TCM decoction pieces allow us to form the following hypothesis.

- *Hypothesis 1* During the COVID-19 pandemic, physicians were more likely to prescribe TCM decoction pieces compared to before the pandemic for both cost-sharing and self-paying visits.

Next, we consider other potential physician responses that could vary by visit payment method. Under the capitated global budget model’s incentive scheme, insurance payments for cost-sharing visits could be treated as costs that draw from the budget fund. In the study region, the number of contracted enrollees with each township hospital did not vary much during the study period. To increase the surplus associated with the fund, physicians could have reduced average insurance payments for cost-sharing visits. Since the insurance payments are positively correlated with total expenditures, we expect that there was a decrease in healthcare expenditures among cost-sharing visits during the pandemic period.

- *Hypothesis 2 *In the period following the COVID-19 outbreak, there was a decrease in total expenditures, drug expenditures, non-drug expenditures and insurance payments among cost-sharing visits, compared to the period before the outbreak.

For self-paying visits, only providing additional non-drug services would be beneficial for physicians due to the implementation of the zero-markup policy. Whether physicians were able to increase the non-drug expenditures during the pandemic mainly depends on the nature of the demand among self-paying visits. If the service level for self-paying visits was determined by patients’ willingness to demand as suggested in previous studies [15, 16], we would not expect a significant increase in non-drug expenditures during the pandemic compared to the period before.

- *Hypothesis 3 *In the period following the COVID-19 outbreak, there were no significant changes in non-drug expenditure among self-paying visits, in comparison with the period before the outbreak.

For most outpatient visits to township hospitals, non-drug services provided include general consultation and basic diagnostic tests. Therefore, there are limited options that physicians can choose to induce more services. Moreover, self-paying rural outpatients are likely to be financially constrained, making them less inclined to seek additional services.

## Data

The dataset was obtained from all township hospitals located in two rural counties within the same prefecture of a southern province in China. The data covered all de-identified outpatient records for two time periods: (1) a pre-pandemic period from January 2019 to December 2019, and (2) a during-pandemic period from March 2020 and February 2021. For each outpatient visit, the dataset recorded the visit date, the attending physician, diagnosis, drugs prescribed and patient characteristics including age, sex, and payment method (cost-sharing or self-paying). The exact amount of total expenditure and drug expenditure, non-drug service expenditure and insurance payments for visits under cost-sharing were also documented.

In the data, the diagnosis for a visit is coded using the International Classification of Diseases, 10th revision (ICD-10). According to World Health Organization guidelines, the ICD10 system is classified into 20 Major Diagnosis Categories (MDCs). Table 1 presents the number and share of outpatient visits before and after the COVID-19 outbreak by MDCs. Although the proportion of respiratory disease visits decreased from 48.9% to 33.7% of the total volume, it remained the most common diagnosis for outpatient visits to township hospitals. The number of visits for other disease categories remained relatively stable. Most of the 18% reduction in outpatient volume following the COVID-19 outbreak was attributed to the decrease in respiratory-related visits.

**Table 1:** Outpatient visits by disease category

Disease category	Before	After
Respiratory	246,943 (48.89%)	139,411 (33.69%)
Infectious	12,414 (2.46%)	11,552 (2.79%)
Neoplasms	653 (0.13%)	804 (0.19%)
Blood	1,008 (0.20%)	1,002 (0.24%)
Metabolic	7,812 (1.55%)	14,502 (3.50%)
Mental	1,486 (0.29%)	1,514 (0.37%)
Nerve	2,714 (0.54%)	3,589 (0.87%)
Eye	3,081 (0.61%)	3,135 (0.76%)
Ear	2,053 (0.41%)	2,049 (0.50%)
Circulatory	30,778 (6.09%)	45,324 (10.95%)
Digestive	40,690 (8.06%)	42,040 (10.16%)
Skin	17,271 (3.42%)	18,837 (4.55%)
Musculoskeletal	35,035 (6.94%)	38,263 (9.25%)
Genitourinary	16,327 (3.23%)	18,157 (4.39%)
Pregnancy	3,187 (0.63%)	3,437 (0.83%)
Congenital	46 (0.01%)	54 (0.01%)
Unclassified	24,522 (4.85%)	19,792 (4.78%)
Injury	17,208 (3.41%)	16,979 (4.10%)
External morbidity	1,391 (0.28%)	1,593 (0.38%)
Health status	9,973 (1.97%)	10,503 (2.54%)
Missing ICD10	30,555 (6.05%)	21,247 (5.13%)
Obs.	505,147 (100%)	413,784 (100%)

Notes: We define outpatient visits from January 1, 2019 to December 31, 2019 as before the COVID-19 outbreak, and the visits from March 1, 2020 to February 28, 2021 as after the outbreak. The unclassified category correspond to ICD10 code R00-R99. Visits without a ICD10 code in the records are categorized as “Missing ICD10”

For visits classified as cost-sharing in the hospital records, patients need to present a health insurance card issued by the local government at outpatient registration. All outpatient visits recorded without the local health insurance card are categorized as self-paying in the hospital records and are ineligible for reimbursement from URRBMI, regardless of the outpatients’ actual insurance status. The policy of URRBMI in the two sample counties remained consistent throughout the study period. In Table 2, we present the personal characteristics of patients with outpatient visits for the full sample, and separately for the subsamples of the cost-sharing and self-paying visits. During the pandemic period, there was a greater reduction in self-paying visits (about 33%) compared with cost-sharing visits (about 9%).

**Table 2:** Personal characteristics of outpatient visits

	Full sample		Cost-sharing		Self-paying	
	Before	After	Before	After	Before	After
Age (%)						
0–19	38.30	27.92	32.13	23.11	49.89	39.85
20–39	11.34	11.51	9.63	9.40	14.56	16.77
40–59	30.53	34.76	35.07	38.20	22.01	26.23
60+	19.83	25.81	23.18	29.29	13.54	17.14
Sex (%)						
Male	48.97	47.78	47.56	46.68	51.64	50.51
Obs.	505,147	413,784	329,688	295,119	175,459	118,655
Sample size	918,931		624,807		294,124	

Panels A and B of Table 3 report outpatient summary statistics: the probability of receiving TCM decoction pieces; total, drug, and non-drug expenditures for cost-sharing and self-pay visits; the number of drugs per prescription; and the average expenditure per drug. We also provide information on insurance payments for cost-sharing visits. It is worth noting that there were visits classified as cost-sharing in hospital records that have zero insurance payments. This occurred due to some visits were made by URRBMI enrollees who presented the health insurance card at registration after they reached the annual coverage limit. Physicians could not directly observe whether enrollees had reached their coverage limit, so they may not know these visits were essentially self-paying unless informed by the URRBMI enrollees. If all enrollees who had reached their annual coverage limit informed their physicians, the physicians would treat their visits as self-paying. In the robustness check, we reclassify these visits as self-paying visits to test whether the empirical findings remain consistent. The actual situation is likely to lie between these two scenarios. To enable the natural logarithm of insurance payments for analysis, we added RMB 0.01 to insurance payments to address the issue of zero values [40].

**Table 3:** Average outpatient experiences

	Before			After			Diff.
	Mean (Std. Dev)	1 st PCTL	99th PCTL	Mean (Std. Dev)	1 st PCTL	99th PCTL	
*Panel A: Cost-sharing Visits*							
Pr(decoction pieces)	0.022			0.035			0.012***
Total Exp.(RMB)	62.43 (49.97)	12.51	270.6	60.28 (55.76)	10.69	291.7	−2.15***
Drug Exp.(RMB)	41.63 (43.93)	2.00	228.3	40.74 (47.70)	1.42	249.1	−0.89***
Non-Drug Exp.(RMB)	20.80 (24.40)	1.78	116.7	19.54 (29.09)	1.02	141.5	−1.26***
Insurance Pay(RMB)	37.59 (32.40)	0	172.3	37.08 (35.49)	0	188.9	−0.51**
# drugs	6.20 (3.51)	1	16	4.94 (3.21)	1	15	1.26***
Exp/drug (RMB)	9.62 (20.21)	0.86	85.02	11.84 (23.02)	0.771	100.8	2.22***
*Panel B: Self-paying Visits*							
Pr(decoction pieces)	0.019			0.033			0.014***
Total Exp.(RMB)	60.36 (102.62)	8.00	422	66.34 (144.2)	3.72	440	5.98***
Drug Exp.(RMB)	41.04 (84.67)	1.23	280	46.29 (126.1)	1.03	298	5.25***
Non-Drug Exp.(RMB)	19.33 (31.68)	0.05	143	20.07 (39.61)	0.05	149.5	0.73***
# drugs	5.09 (3.32)	1	15	4.31 (3.11)	1	15	0.62***
Exp/drug (RMB)	15.86 (55.89)	0.64	280.0	19.59 (61.44)	0.61	228.0	3.73***

Notes: PCTL is the abbreviation for percentile. 1 RMB=0.145 US dollars in 2020. Differences in sample means before and after the COVID-19 outbreak were compared using a two-proportion z-test for binary variables or a two-sample t-test for continuous variables. ***, ** and * represent statistical significance at 0.001, 0.01 and 0.05 level, respectively

Table 3 shows that the probability of prescribing TCM decoction pieces increased following the COVID-19 outbreak. Health expenditures among cost-sharing visits decreased significantly, while expenditures among self-paying visits increased significantly. The number of drugs per prescription decreased during the pandemic, whereas the average expenditure per drug increased. The raw average differences presented in Table 3 are only suggestive, as they do not account for important differences in patient characteristics and disease categories between the before and after COVID periods.

## Empirical approach

The following specification is used to examine physician practice patterns before and during the COVID-19 pandemic:

1 $$\begin{aligned} Y_{i j t}= \beta Covid_{t} +\textbf{X}_{\textbf{i}}+\gamma _{j}+\gamma _{t}+\varepsilon _{i j t}. \end{aligned}$$

For patient *i* whose attending physician is *j* at time (month) *t*, the dependent variable is whether the prescription from the visit contains TCM decoction pieces, or the visit’s total expenditures as well as drug expenditures and non-drug services expenditures. $Covid_{t}$ is a dummy variable that equals 1 if the outpatient visit occurred after the COVID-19 outbreak (during the pandemic, from March 2020 to February 2021), and equals 0 if the outpatient visit happened before the outbreak(before the pandemic, from January to December 2019). $\textbf{X}_{\textbf{i}}$ are patient characteristics including age, sex, and dummies for the 20 MDCs to control the diagnosis codes. $\gamma _{j}$ captures the physician fixed effects of 375 physicians in the sample, and $\gamma _{t}$ controls the month fixed effects. We cluster the robust standard errors at the physician level. The coefficients of interests in Equation (1) is $\beta$, which estimates the effect of physician responses to the COVID-19 pandemic on care provision.

Different regression models are employed based on the nature of the dependent variable. When the dependent variable is whether a prescription from a visit contained TCM decoction pieces, we use both ordinary least squares (OLS) and logit models. Because not all physicians prescribe TCM decoction pieces, we restricted the analysis to patients treated by physicians who ever prescribed them during the study period. Physician-level heterogeneity was assessed by stratifying patients according to their attending physicians’ pre-pandemic share of patients receiving TCM decoction prescriptions.

For expenditure-related dependent variables, we used generalized linear models (GLM) with a log link function and Gamma distribution to account for the skewed nature of health expenditure data. This approach is widely used in modeling health expenditures as it effectively handles non-normal distributions and allows for proportional changes, providing a more robust fit [41–44].

Additionally, we use this framework to decompose changes in drug expenditure into two channels: (i) the number of drugs per prescription (quantity) and (ii) the average expenditure per drug (price). The number of drugs per prescription is treated as a count outcome and estimated using GLM with a log link function and Poisson distribution; OLS results are reported as a benchmark. The average expenditure per drug is analyzed using OLS and GLM consistent with our approach to other expenditure outcomes.

## Results

### Physician response in prescribing TCM decoction pieces

Table 4 presents the OLS and the logit estimates for whether a prescription from a visit contains TCM decoction pieces.The results indicate that, following the COVID-19 outbreak, there was a significant increase of more than 1 percentage point, or approximately 1.5 times the odds of prescribing TCM decoction pieces for both cost-sharing and self-paying visits, consistent with Hypothesis 1. However, the impact of this increase on patients’ medication regimens remains modest, as prescriptions containing decoction pieces still comprised only a small percentage of total prescriptions.

**Table 4:** Effects of physician response on the prescribing of TCM decoction pieces

	Cost-sharing visits		Self-paying visits	
	OLS Coef.	Logit Odds ratio	OLS Coef.	Logit Odds ratio
Covid	0.016***	1.446***	0.012***	1.578**
	(0.003)	(0.173)	(0.002)	(0.212)
Characteristics	Y	Y	Y	Y
Diagnosis	Y	Y	Y	Y
Physician FE	Y	Y	Y	Y
Month FE	Y	Y	Y	Y
Sample size	472,234	472,234	228,877	228,877
Pseudo/Adj. R^2^	0.239	0.224	0.180	0.186

Notes: In all the regressions, we control for patient demographic characteristics for each visit and the diagnosis in Major Disease Category indicators. ***, ** and * represent statistical significance at 0.001, 0.01 and 0.05 level, respectively. Robust standard errors are clustered at the physician level. Pseudo R^2^ is reported for logit models

Table 5 presents the heterogeneous effects on prescribing TCM decoction pieces by physicians’ baseline prescribing share. Odds ratios are of similar magnitude across strata. In marginal terms, patients treated during the COVID-19 period were about 5.3 percentage points more likely to receive TCM decoction pieces when their attending physician’s baseline share was greater than 0.10; the increase was 2.5 percentage points for physicians with a baseline share between 0.05 and 0.10, and only 0.9 percentage points when prescribing was only occasional at baseline (less than 0.05). As expected, this pattern suggests the shock amplified existing practice styles rather than converting low users into regular prescribers.

**Table 5:** Heterogeneous effects on TCM decoction-piece prescribing by physician baseline share of patients receiving TCM decoction pieces

	0$\varvec{<}$Share$\varvec{\le }$0.05		0.05$\varvec{<}$Share$\varvec{\le }$0.1		Share$\varvec{>}$0.1	
	OLS Coef.	Logit Odds ratio	OLS Coef.	Logit Odds ratio	OLS Coef.	Logit Odds ratio
Covid	0.009***	1.472***	0.025***	1.439***	0.053***	1.410***
	(0.002)	(0.283)	(0.008)	(0.269)	(0.015)	(0.215)
Characteristics	Y	Y	Y	Y	Y	Y
Diagnosis	Y	Y	Y	Y	Y	Y
Physician FE	Y	Y	Y	Y	Y	Y
Month FE	Y	Y	Y	Y	Y	Y
Sample size	576,255	576,255	70,683	70,683	54,178	54,178
Pseudo/Adj. R^2^	0.232	0.204	0.225	0.198	0.211	0.187

Notes: In all the regressions, we control for patient demographic characteristics for each visit and the diagnosis in Major Disease Category indicators. ***, ** and * represent statistical significance at 0.001, 0.01 and 0.05 level, respectively. Robust standard errors are clustered at the physician level. Pseudo R^2^ is reported for logit models

### Physician response in healthcare expenditures

Table 6 reports the results from both OLS and GLM models. For cost-sharing visits, total expenditures, drug expenditures, non-drug service expenditures, and insurance payments were all reduced during the COVID-19 pandemic compared to the pre-pandemic period. Specifically, total expenditures decreased by approximately 7.1%, and insurance payments fell by around 4.5%. Meanwhile, among self-paying patients, total expenditures declined by a smaller margin of 3.1%, while non-drug service expenditures showed no significant change.

**Table 6:** Effects of physician response on expenditures

	Cost-sharing visits		Self-paying visits	
	OLS Coef.	GLM Coef.	OLS Coef.	GLM Coef.
*DV = Total Expenditures*				
Covid	−4.92***	−0.074***	−1.25	−0.031*
	(0.752)	(0.011)	(1.111)	(0.015)
Characteristics	Y	Y	Y	Y
Diagnosis	Y	Y	Y	Y
Physician FE	Y	Y	Y	Y
Month FE	Y	Y	Y	Y
Adj.R^2^	0.212		0.315	
*DV = Drug Expenditures*				
Covid	−3.89***	−0.078***	−0.72	−0.036*
	(0.699)	(0.015)	(1.048)	(0.019)
Characteristics	Y	Y	Y	Y
Diagnosis	Y	Y	Y	Y
Physician FE	Y	Y	Y	Y
Month FE	Y	Y	Y	Y
Adj.R^2^	0.291		0.307	
*DV = Non-drug Expenditures*				
Covid	−1.05***	−0.063***	−0.53	−0.032
	(0.249)	(0.011)	(0.284)	(0.021)
Characteristics	Y	Y	Y	Y
Diagnosis	Y	Y	Y	Y
Physician FE	Y	Y	Y	Y
Month FE	Y	Y	Y	Y
Adj.R^2^	0.165		0.235	
*DV = Insurance Payments*				
Covid	−2.18***	−0.046***		
	(0.479)	(0.012)		
Characteristics	Y	Y		
Diagnosis	Y	Y		
Physician FE	Y	Y		
Month FE	Y	Y		
Adj.R^2^	0.163			
Sample size	624,807	624,807	294,124	294,124

Notes: In all the regressions, we control for patient demographic characteristics for each visit and the diagnosis in Major Disease Category indicators. ***, ** and * represent statistical significance at 0.001, 0.01 and 0.05 level, respectively. Robust standard errors are clustered at the physician level

### Heterogeneous response by disease categories

Respiratory diseases are the most common reason for outpatient care, and patients with certain respiratory symptoms may have avoided seeking care due to COVID-19 restrictions. To better understand changes in physician practices, we separately estimated regressions for respiratory and non-respiratory diseases based on Equation (1). The results, presented in Tables 7 and 8, suggest that for cost-sharing visits related to respiratory disease following the COVID-19 outbreak, total expenditures decreased by 4.7 log points (approximately 4.6%) and insurance payments dropped by 2.6 log points (around 2.6%). Self-paying visits related to respiratory diseases experienced a larger reduction in total expenditures, with a decrease of 6.4 log points (about 6.2%). These findings suggest that respiratory-related outpatient visits during the pandemic may have required less intensive treatment compared to pre-pandemic visits.

**Table 7:** Effects of physician responses on expenditures, respiratory disease

	Cost-sharing visits		Self-paying visits	
	OLS Coef.	GLM Coef.	OLS Coef.	GLM Coef.
*DV = Total Expenditures*				
Covid	−2.68***	−0.047***	−3.20***	−0.064***
	(0.787)	(0.014)	(0.726)	(0.014)
Characteristics	Y	Y	Y	Y
Physician FE	Y	Y	Y	Y
Month FE	Y	Y	Y	Y
Adj.R^2^	0.227		0.195	
*DV = Drug Expenditures*				
Covid	−1.68**	−0.031*	−2.38***	−0.063**
	(0.710)	(0.016)	(0.657)	(0.020)
Characteristics	Y	Y	Y	Y
Physician FE	Y	Y	Y	Y
Month FE	Y	Y	Y	Y
Adj.R^2^	0.314		0.256	
*DV = Non-drug Expenditures*				
Covid	−1.00***	−0.062***	−0.82***	−0.055***
	(0.308)	(0.014)	(0.204)	(0.012)
Characteristics	Y	Y	Y	Y
Physician FE	Y	Y	Y	Y
Month FE	Y	Y	Y	Y
Adj.R^2^	0.158		0.141	
*DV = Insurance Payments*				
Covid	−1.07**	−0.026**		
	(0.452)	(0.012)		
Characteristics	Y	Y		
Physician FE	Y	Y		
Month FE	Y	Y		
Adj.R^2^	0.154			
Sample size	262,899	262,899	123,455	123,455

Notes: In all the regressions, we control for patient demographic characteristics for each visit and the diagnosis in Major Disease Categories. ***, **, and * represent statistical significance at 0.001, 0.01 and 0.05 level, respectively. Robust standard errors are clustered at the physician level

**Table 8:** Effects of physician responses on expenditures, non-respiratory disease

	Cost-sharing visits		Self-paying visits	
	OLS Coef.	GLM Coef.	OLS Coef.	GLM Coef.
*DV = Total Expenditures*				
Covid	−6.26***	−0.093***	−0.10	−0.017
	(0.992)	(0.012)	(1.580)	(0.015)
Characteristics	Y	Y	Y	Y
Diagnosis	Y	Y	Y	Y
Physician FE	Y	Y	Y	Y
Month FE	Y	Y	Y	Y
Adj.R^2^	0.217		0.313	
*DV = Drug Expenditures*				
Covid	−5.24***	−0.113***	0.22	−0.003
	(0.939)	(0.015)	(1.49)	(0.021)
Characteristics	Y	Y	Y	Y
Diagnosis	Y	Y	Y	Y
Physician FE	Y	Y	Y	Y
Month FE	Y	Y	Y	Y
Adj.R^2^	0.297		0.306	
*DV = Non-drug Expenditures*				
Covid	−1.02***	−0.062***	−0.32	−0.025
	(0.270)	(0.011)	(0.340)	(0.014)
Characteristics	Y	Y	Y	Y
Diagnosis	Y	Y	Y	Y
Physician FE	Y	Y	Y	Y
Month FE	Y	Y	Y	Y
Adj.R^2^	0.162		0.238	
*DV = Insurance Payments*				
Covid	−2.95***	−0.061***		
	(0.616)	(0.012)		
Characteristics	Y	Y		
Diagnosis	Y	Y		
Physician FE	Y	Y		
Month FE	Y	Y		
Adj.R^2^	0.168			
Sample size	361,908	361,908	170,669	170,669

Notes: In all the regressions, we control for patient demographic characteristics for each visit and the diagnosis in Major Disease Category indicators. ***, ** and * represent statistical significance at 0.001, 0.01 and 0.05 level, respectively. Robust standard errors are clustered at the physician level

Table 8 provides a contrasting perspective. Unlike the findings in Table 7, total expenditures for self-paying non-respiratory visits did not change significantly during the pandemic compared to the pre-pandemic period. This suggests that the average severity of illness for non-respiratory visits remained relatively stable throughout the study period. Taken together, the results from Tables 7 and 8 indicate that the overall decrease in expenditures for self-paying visits (as shown in Table 6) was primarily driven by reductions in expenditures for respiratory-related self-paying visits. Furthermore, there was no notable increase in non-drug service expenditures among self-paying visits after the COVID-19 outbreak.

For non-respiratory cost-sharing visits, there was an approximate 9% decrease in total expenditures and a 6% decrease in insurance payments. The magnitude of the expenditure reduction for non-respiratory cost-sharing visits appears greater than for respiratory-related visits. These reductions in healthcare utilization were at least partially attributable to physician-induced demand. Overall, the results presented in Tables 6, 7, and 8 align with Hypotheses 2 and 3.

### Decomposing the channels leading to reduced drug expenditures

Table 9 reports physician responses along two margins—the number of drugs per prescription and the average expenditure per drug—separately for respiratory and non-respiratory diagnoses and by payer type. Among patients with respiratory diagnoses, the number of drugs per prescription declined during the pandemic. The average expenditure per drug rose slightly, but not enough to increase total prescription cost per visit as shown in Table 7. These patterns are similar for both cost-sharing and self-pay visits.

For non-respiratory diagnoses, cost-sharing patients experienced declines in both the number of drugs per prescription and the average expenditure per drug, implying a clear reduction in drug spending. Among self-pay patients, the number of drugs per prescription fell by a smaller amount, and the average expenditure per drug did not change significantly.

**Table 9:** Effects of physician responses on the number of drugs on a prescription and expenditures per drug

	Cost-sharing visits		Self-paying visits	
	OLS Coef.	GLM Coef.	OLS Coef.	GLM Coef.
*DV = # drugs*-*respiratory disease*				
Covid	−1.395***	−0.225***	−1.077***	−0.198***
	(0.060)	(0.010)	(0.057)	(0.013)
Characteristics	Y	Y	Y	Y
Physician FE	Y	Y	Y	Y
Month FE	Y	Y	Y	Y
Adj.R^2^	0.221		0.168	
Sample size	262,809	262,809	123,455	123,455
*DV = Exp/drug-respiratory disease*				
Covid	1.269***	0.215***	0.764***	0.144***
	(0.111)	(0.024)	(0.092)	(0.018)
Characteristics	Y	Y	Y	Y
Physician FE	Y	Y	Y	Y
Month FE	Y	Y	Y	Y
Adj.R^2^	0.206		0.170	
Sample size	262,809	262,809	123,455	123,455
*DV = # drugs*-*non-respiratory disease*				
Covid	−0.554***	−0.111***	−0.230***	−0.060***
	(0.061)	(0.012)	(0.056)	(0.014)
Characteristics	Y	Y	Y	Y
Diagnosis	Y	Y	Y	Y
Physician FE	Y	Y	Y	Y
Month FE	Y	Y	Y	Y
Adj.R^2^	0.269		0.277	
Sample size	361,908	361,908	170,669	170,669
*DV = Exp/drug-non-respiratory disease*				
Covid	−1.263***	−0.156***	−0.661	−0.009
	(0.309)	(0.016)	(1.450)	(0.012)
Characteristics	Y	Y	Y	Y
Diagnosis	Y	Y	Y	Y
Physician FE	Y	Y	Y	Y
Month FE	Y	Y	Y	Y
Adj.R^2^	0.244		0.306	
Sample size	361,908	361,908	170,669	170,669

Notes: In all regressions, we control for visit-level patient demographics. In specifications restricted to non-respiratory visits, we additionally include Major Disease Category indicators. ***, ** and *represent statistical significance at 0.001, 0.01 and 0.05 level, respectively. Robust standard errors are clustered at the physician level.

### Robustness check

So far, we have assumed that visits by URRBMI enrollees who had already reached their coverage limit were not disclosed to attending physicians. Consequently, these visits were recorded as cost-sharing in hospital records, even though enrollees paid the full amount out of pocket. There were 19,202 such visits in the pre-pandemic period and 15,486 during the pandemic. If attending physicians had complete information about which enrollees had reached their limit, they might have treated these visits as self-paying. In practice, physicians likely know patients’ coverage status to some extent. As a robustness check, we reclassify these visits as self-pay and re-estimate the models; the results are reported in Table 10. As a second check, we replace physician fixed effects with hospital fixed effects and cluster robust standard errors at the hospital level; the results are presented in Table 11. In both cases, the estimates closely mirror the main results in Table 6, reinforcing the robustness of our findings.

**Table 10:** Robust check: reclassified payment method of outpatient visits

	Cost-sharing visits		Self-paying visits	
	OLS Coef.	GLM Coef.	OLS Coef.	GLM Coef.
*DV = Total Expenditures*				
Covid	−5.090***	−0.077***	−1.131	−0.029*
	(0.743)	(0.011)	(1.081)	(0.014)
Characteristics	Y	Y	Y	Y
Diagnosis	Y	Y	Y	Y
Physician FE	Y	Y	Y	Y
Month FE	Y	Y	Y	Y
Adj.R^2^	0.208		0.313	
*DV = Drug Expenditures*				
Covid	−4.031***	−0.080***	−0.602	−0.033
	(0.685)	(0.015)	(1.023)	(0.020)
Characteristics	Y	Y	Y	Y
Diagnosis	Y	Y	Y	Y
Physician FE	Y	Y	Y	Y
Month FE	Y	Y	Y	Y
Adj.R^2^	0.286		0.306	
*DV = Non-drug Expenditures*				
Covid	−1.062***	−0.063***	−0.533	−0.028
	(0.251)	(0.011)	(0.282)	(0.019)
Characteristics	Y	Y	Y	Y
Diagnosis	Y	Y	Y	Y
Physician FE	Y	Y	Y	Y
Month FE	Y	Y	Y	Y
Adj.R^2^	0.167		0.221	
*DV = Insurance Payments*				
Covid	−2.620***	−0.058***		
	(0.533)	(0.012)		
Characteristics	Y	Y		
Diagnosis	Y	Y		
Physician FE	Y	Y		
Month FE	Y	Y		
Adj.R^2^	0.195			
Sample size	590,119	590,119	328,812	328,812

Notes: In all the regressions, we control for patient demographic characteristics for each visit and the diagnosis in 20 Major Disease Category indicators. ***, ** and * represent statistical significance at 0.001, 0.01 and 0.05 level, respectively. Robust standard errors are clustered at the physician level

**Table 11:** Robust check: models with hospital fixed effects

	Cost-sharing visits		Self-paying visits	
	OLS Coef.	GLM Coef.	OLS Coef.	GLM Coef.
*DV = Total Expenditures*				
Covid	−4.892***	−0.071***	−0.030	−0.016
	(1.214)	(0.017)	(1.498)	(0.022)
Characteristics	Y	Y	Y	Y
Diagnosis	Y	Y	Y	Y
Hospital FE	Y	Y	Y	Y
Month FE	Y	Y	Y	Y
Adj.R^2^	0.164		0.179	
*DV = Drug Expenditures*				
Covid	−3.739***	−0.070**	0.267	−0.018
	(1.071)	(0.022)	(1.534)	(0.031)
Characteristics	Y	Y	Y	Y
Diagnosis	Y	Y	Y	Y
Hospital FE	Y	Y	Y	Y
Month FE	Y	Y	Y	Y
Adj.R^2^	0.242		0.161	
*DV = Non-drug Expenditures*				
Covid	−1.153**	−0.065**	−0.297	−0.017
	(0.407)	(0.023)	(0.512)	(0.028)
Characteristics	Y	Y	Y	Y
Diagnosis	Y	Y	Y	Y
Hospital FE	Y	Y	Y	Y
Month FE	Y	Y	Y	Y
Adj.R^2^	0.068		0.143	
*DV = Insurance Payments*				
Covid	−2.17**	−0.043**		
	(0.764)	(0.017)		
Characteristics	Y	Y		
Diagnosis	Y	Y		
Hospital FE	Y	Y		
Month FE	Y	Y		
Adj.R^2^	0.132			
Sample size	624,807	624,807	294,124	294,124

Notes: In all the regressions, we control for patient demographic characteristics for each visit and the diagnosis in 20 Major Disease Category indicators. ***, ** and * represent statistical significance at 0.001, 0.01 and 0.05 level, respectively. Robust standard errors are clustered at the hospital level

## Discussion and conclusions

This paper examines how physicians adjusted their practice patterns in a rural primary care setting in China by leveraging the financial pressures hospitals faced during the COVID-19 pandemic. We find that, relative to the pre-pandemic period, physicians significantly increased the prescribing of TCM decoction pieces during the pandemic, with the increase concentrated among physicians who were already regular prescribers prior to the pandemic.

We also show that, following the COVID-19 outbreak, average insurance payments per visit fell by about 5%, consistent with incentives embedded in the capitated global-budget model. These lower draws on the capitated budget fund were achieved through a combination of fewer drugs per prescription, prescribing lower-cost drugs (lower expenditure per drug), and providing fewer services.

In contrast, we find no significant change in service volume for self-paying outpatients, consistent with these visits being largely patient-driven and leaving little scope for physician-induced demand. Because physicians play a central role in care delivery, incorporating physician agency into policy design is critical for achieving intended objectives. Our findings offer insights for policy development in other low- and middle-income countries with similar physician remuneration structures.

China has long faced criticism over misaligned financial incentives in prescription drugs, which contributed to physician-induced demand and overprescribing. Since the drug reform initiated in 2009 and fully implemented in 2017, the profit motive tied to most outpatient drug dispensing has largely been removed. This paper examines physician agency in this post–drug-markup era, in which public primary care hospitals are prohibited from charging markups on most drugs. Our results indicate that, in rural outpatient care, total health expenditures—including drug spending—were effectively contained following the drug reform and the introduction of managed-care elements such as a capitated global-budget model. However, capitation rates for township hospitals and per-resident reimbursement limits must be set carefully; current URRBMI standards may not provide adequate coverage for rural residents. Under stress conditions such as COVID-19, URRBMI enrollees may face an elevated risk of underprovision due to physician-agency responses. Additional government investment may be needed to strengthen URRBMI coverage.

This study has several limitations. First, our data come from township hospitals in a less economically developed region; substantial heterogeneity likely exists between rural and urban populations and across areas with different levels of economic development. Consequently, our findings on physician agency may be only suggestive for other settings. Second, the dataset contains no quality-of-care measures. Although we document changes in the quantity of care during the pandemic, we cannot assess how these shifts affected care quality or patient outcomes. Related evidence from Nigeria indicates that potential quality indicators—such as adherence to clinical guidelines and completion of routine procedures—deteriorated during the early months of COVID-19 [45]. Third, itemized drug costs are unavailable, preventing a more granular analysis of the pathways through which physicians influenced drug expenditures. Finally, patient-level controls are limited to age and sex and do not capture socioeconomic status or illness severity; changes in these dimensions before versus during the pandemic could confound estimated physician responses. We view this risk as limited for two reasons. First, our dataset covers the universe of outpatient visits in two rural counties, and the underlying populations were stable over the 24-month study period. Second, trends in socioeconomic status or illness severity would likely affect cost-sharing and self-pay visits similarly, yet we observe markedly different physician responses across these groups, suggesting that at least part of the difference reflects physician agency. Nevertheless, because our identification relies on a before–after design, residual confounding cannot be fully ruled out.

While the policy implications of this paper are most relevant to China and other emerging economies with similar financial incentive schemes for primary care physicians, they may not generalize to developed countries with different physician compensation models. Nonetheless, the issue of physician agency arises to varying degrees across different payment systems and should not be overlooked in policy design. This paper represents an initial step in exploring physician agency under the influence of the COVID-19 pandemic and the current policy context, using a sample of primary care physicians working in rural township hospitals. Future research is needed to provide a more comprehensive view across different healthcare settings and incentive schemes in various geographic areas.

## Data Availability

The data used in this research article are regulated by the government and require official approval from either the National Health Commission of the PRC or provincial Health Commission for access and use.

## Footnotes

1 Data source: https://www.ipe.org.cn/MapGZBD/GZBDMap.html.

2 China has five levels of local government. The study counties are under the same prefecture.
